# Luminance- and Texture-Defined Information Processing in School-Aged Children with Autism

**DOI:** 10.1371/journal.pone.0078978

**Published:** 2013-10-24

**Authors:** Jessica B. Rivest, Boutheina Jemel, Armando Bertone, Michelle McKerral, Laurent Mottron

**Affiliations:** 1 University of Montreal Center of Excellence for Pervasive Developmental Disorders (CETEDUM), Montreal, Quebec, Canada; 2 Centre de Recherche en Neuropsychologie et Cognition (CERNEC) and Department of Psychology, University of Montreal, Montreal, Quebec, Canada; 3 Research Laboratory in Neuroscience and Cognitive Electrophysiology, Rivière-des-Prairies Hospital, University of Montreal, Montreal, Quebec, Canada; Vanderbilt University, United States of America

## Abstract

According to the complexity-specific hypothesis, the efficacy with which individuals with autism spectrum disorder (ASD) process visual information varies according to the extensiveness of the neural network required to process stimuli. Specifically, adults with ASD are less sensitive to texture-defined (or second-order) information, which necessitates the implication of several cortical visual areas. Conversely, the sensitivity to simple, luminance-defined (or first-order) information, which mainly relies on primary visual cortex (V1) activity, has been found to be either superior (static material) or intact (dynamic material) in ASD. It is currently unknown if these autistic perceptual alterations are present in childhood. In the present study, behavioural (threshold) and electrophysiological measures were obtained for static luminance- and texture-defined gratings presented to school-aged children with ASD and compared to those of typically developing children. Our behavioural and electrophysiological (P140) results indicate that luminance processing is likely unremarkable in autistic children. With respect to texture processing, there was no significant threshold difference between groups. However, unlike typical children, autistic children did not show reliable enhancements of brain activity (N230 and P340) in response to texture-defined gratings relative to luminance-defined gratings. This suggests reduced efficiency of neuro-integrative mechanisms operating at a perceptual level in autism. These results are in line with the idea that visual atypicalities mediated by intermediate-scale neural networks emerge before or during the school-age period in autism.

## Introduction

In addition to atypical social and communication and repetitive/stereotyped behaviours and interests, individuals on the autism spectrum also present alterations in visuo-perceptual information processing [1,2]. One of the most replicated findings is the modified hierarchy in the perception for compound visual patterns, or a local bias when processing visual information (see [3] for review). Individuals with ASD often manifest superior performance on visual tasks in which global precedence typically slows down or precludes detection of local aspects, such as the Embedded Figures test [4,5], Block Design task [6,7], and visual search tests [8-11]. There are two main accounts for this characteristic performance, the Weak Central Coherence (WCC) theory [12], and the Enhanced Perceptual Functioning (EPF) model [13,14]. The WCC hypothesis states that impairment of top down influence on incoming information is either impaired or reduced in its default application. When applied to compound or complex information, where top down influence is represented by global precedence, WCC would favor a local perceptual perspective. In contrast, the EPF account states that low-level perception is over-developed in autism, where high-level, cognitive functions are more dependent on perceptual processes, and where global processing is intact, but optional. 

In addition to the local/global distinction, autistic perception has also been dichotomized within the context of static versus dynamic visual information perception. The dorsal stream hypothesis proposes that dynamic information processing is affected in ASD [15], accounting for reduced sensitivity to complex types of dynamic information such as global and biological motion [16-20]. However, this theory has been challenged by findings of intact processing of more simple types of motion in autism [21,22], as well as of problems integrating complex static information, suggesting a ventral stream dysfunction [18,19]. 

Another distinction that has been used in the study of visual information processing in ASD is that between simple, luminance-defined (first-order or FO) and complex, texture-defined (second-order or SO) visual cues. Accordingly, FO and SO attributes rely on distinct neural networks in the visual brain [23-25]. More specifically, luminance stimuli are mediated by striate mechanisms (i.e., V1), whereas texture stimuli require an additional step of processing and are mediated by extrastriate mechanisms (i.e., V1, V2, V3). Several lines of evidence support the idea that luminance and texture perception operate at different levels of complexity along the visual pathways (but see Ashida et al., 2007 [26] for a discussion about conflicting evidence), namely electrophysiological research in animals [27] and humans [28,29], neuropsychological reports of brain-damaged patients [30,31], and functional magnetic resonance imaging (fMRI) studies [32-34]. When measured in autistic adults, autistic participants show either superior (static) or intact (dynamic) FO sensitivity, while sensitivity to SO information, whether static or dynamic, is inferior when compared to neurotypical observers [35,36]. The behavioural dissociation evident in the static domain has been interpreted as a perceptual signature that is specific to autism, as it has not been observed in other neurodevelopmental conditions such as Fragile X syndrome [37], in individuals having suffered from mild traumatic brain injury [38,39], or during non-pathological aging [40]. This autistic perceptual signature gave rise to the complexity-specific hypothesis [41] which stipulates a decreased efficiency at processing visual information in autism when cooperation between functional visual regions is necessary (i.e., interactions between V1 and extrastriate areas V2, V3), concomitant with an intact or increased efficiency at processing stimuli mainly extracted within the primary visual cortex (V1). In terms of the underlying neural alterations that could account for this dissociation in performance, abnormal lateral and/or feedback connectivity within low-level visual areas was suggested [36]. The complexity-specific hypothesis is an alternative to the “dorsal stream vulnerability” hypothesis, as it is argued that autistics will show impairments of both ventral and dorsal stream-mediated visual processing whenever visual tasks involve high levels of analysis (i.e., collaboration of cortical areas) (see Bertone and Faubert, 2006 [41] for a comprehensive discussion). 

The complexity-specific hypothesis and its tenets are compatible with those of other studies [42,43], and has influenced models of autistic cognition. Notably, the EPF model integrated some of the complexity-specific assumptions into its principles of autistic perception [14]. Nevertheless, it is currently unknown whether the FO-SO dissociation is present in young individuals with autism, as the original studies focused exclusively on adults [35,36]. Given the differential maturation of visual mechanisms mediating FO and SO information throughout childhood [44,45], determining whether atypical FO and SO processing in autism emerges early or late in development is a crucial issue for perceptual theories of autism. The aim of this study was thus to investigate FO and SO processing in autistic children, using both behavioural (psychophysics) and electrophysiological (visual evoked potentials: VEPs) approaches. Current source density (CSD) analysis was performed on the electrophysiological data given the topographic premises of the complexity-specific hypothesis (i.e., striate versus extrastriate functioning). 

## Materials and Methods

### Ethics Statement

Prior to the beginning of the experiment, written informed consent was obtained from all children and their parents under a protocol that was approved by the research ethics committees of both Rivière-des-Prairies and Ste-Justine Hospitals in Montreal, Canada.

### Participants

The autistic (AUT) group was comprised of school-aged children (6 to 11 years-old) having received a diagnosis of Autistic Disorder from a multi-disciplinary clinical team, most of them at the Specialized Clinic of Pervasive Developmental Disorders of Rivière-des-Prairies Hospital (Montreal, Canada). The diagnosis of autism disorder stricto sensu (not ASD) was based the combination of Autism Diagnostic Interview-Revised (ADI-R) [46], the Autism Diagnostic Observation Schedule-General (ADOS-G) *[47], and clinical judgments of experts in the field*. Twenty-four autistic children were initially recruited, but the final group comprised 17 children in total (13 and 15 children for the psychophysical and electrophysiological tasks, respectively). Of those who were not included, two could not complete the cognitive assessments, eight could not adequately perform the psychophysical task due to poor eye fixation, fatigue, or hyperactivity/impulsivity. One child was also excluded from the psychophysical analyses because of important response inconsistencies during the task. With regard to electroencephalography (EEG), six children were not compliant with the procedure, and data from one child were discarded because of movement artifacts and poor eye fixation. 

Fourteen children out of 17 within the final clinical group met full ADI-R and ADOS-G criteria for Autistic Disorder. One child scored above the ADI-R and was subthreshold for the ADOS-G cut-offs on the communication and combined social/communication domains, and another child scored above the ADOS-G and was subthreshold for the ADI-R cut-offs in the reciprocal social interactions domain, while both still positive for an expert Autistic Disorder diagnostic. The remaining child was only administered an ADI-R and was positive on this instrument. On the 17 autistic children, six had a speech delay (average age of two-word sentence = 45 months) and two had not reached this milestone at the time of the ADI-R. Data on speech milestone were missing for two children and the remaining seven children had no significant speech delay (average age of two-word sentence = 21.86 months), but all nine showed other autistic language characteristics (for ex., echolalia, stereotyped utterances, pronominal reversal). With respect to clinical comorbidities, two autistic children also received a diagnosis of Attention-Deficit/Hyperactivity Disorder and one autistic child met criteria for a mood disorder. None of the autistic participants had indications of a recognizable neurological or genetic disorder (e.g., epilepsy, Fragile X syndrome).

Twenty-three typically developing children were recruited through advertising in the general population, and assigned to the control group (TYP). They were screened with an in-house questionnaire completed by their parents and were excluded if they had any neurological, developmental or psychiatric conditions, or a first- or second-degree familial history of ASD or schizophrenia. Psychophysical data from one typical child were discarded because of important response inconsistencies during the task. Another child was excluded for both tasks because of accidental discovery of abnormal EEG activity and had been referred for additional examination in neurology according to our ethical protocol. Finally, three typical children only performed the psychophysical task because they failed to come back for a second visit to the research laboratory. The final control group consisted of 21 and 19 individuals for the psychophysical and electrophysiological tasks, respectively. 

The groups were matched for chronological age and intellectual quotient using the Raven Progressive Matrices (RPM)*, a measure of fluid intelligence [48]. Their verbal receptive abilities were assessed with the Peabody Picture Vocabulary Test-Revised (PPVT-R*) [49]. *As compared to typical children, autistic children scored significantly lower on the PPVT-R for the* electrophysiological part of the experiment. This is consistent with language difficulties being a core feature of autism. Socio-demographic characteristics are presented in Table 1. All children in both groups had normal or corrected-to-normal vision, as measured by visual acuity scores within the normality range using Lea symbols [50]. This test of visual acuity is recommended in populations with low verbal abilities. At the time of the testing, six children were on medication (methylphenidate for two autistic children; both methylphenidate and atomoxetine for one autistic child; both amphetamine/dextroamphetamine and clonidine for one autistic child; both risperidone and atomoxetine for on autistic child; atomoxetine for one typical child who had no psychological diagnosis). 

**Table 1:** Description of the participants.

**Psychophysical Task**			
	**Typical Group**	**Autism Group**	***t*-tests**
**Gender**	19 Males/ 2 Females	12 Males/ 1 Female	-----
**Chronological age- years** Mean + SD (Range)	8.89 + 1.48 (6.17- 11)	9.17 + 1.45 (6.17- 11)	*p* = 0.59
**RMP percentiles** Mean + SD (Range)	80.76 + 17.9 (50-99)	76.62 + 24.87 (28-97)	*p* = 0.58
**PPVT-R- standard scores** Mean + SD (Range)	122.43 + 12.78 (101-143)	113.15 + 19.18 (78-143)	*p* = 0.10
	**Typical Group**	**Autism Group**	***t*-tests**
**Gender**	17 Males/ 2 Females	14 Males/ 1 Female	-----
**Chronological age- years** Mean + SD (Range)	9.07 + 1.22 (7.08- 11)	9.46 + 1.07 (7.25-11)	*p* = 0.34
**RMP percentiles** Mean + SD (Range)	80.84 + 17.81 (50-99)	64.73 + 35.91 (10-97)	*p* =0.13
**PPVT-R standard scores** Mean + SD (Range)	120.58 + 12.66 (101-143)	100.47 + 31.84 (39-143)	*p* < 0.05

Electrophysiological Task

### Visual Stimuli

**Figure 1:**
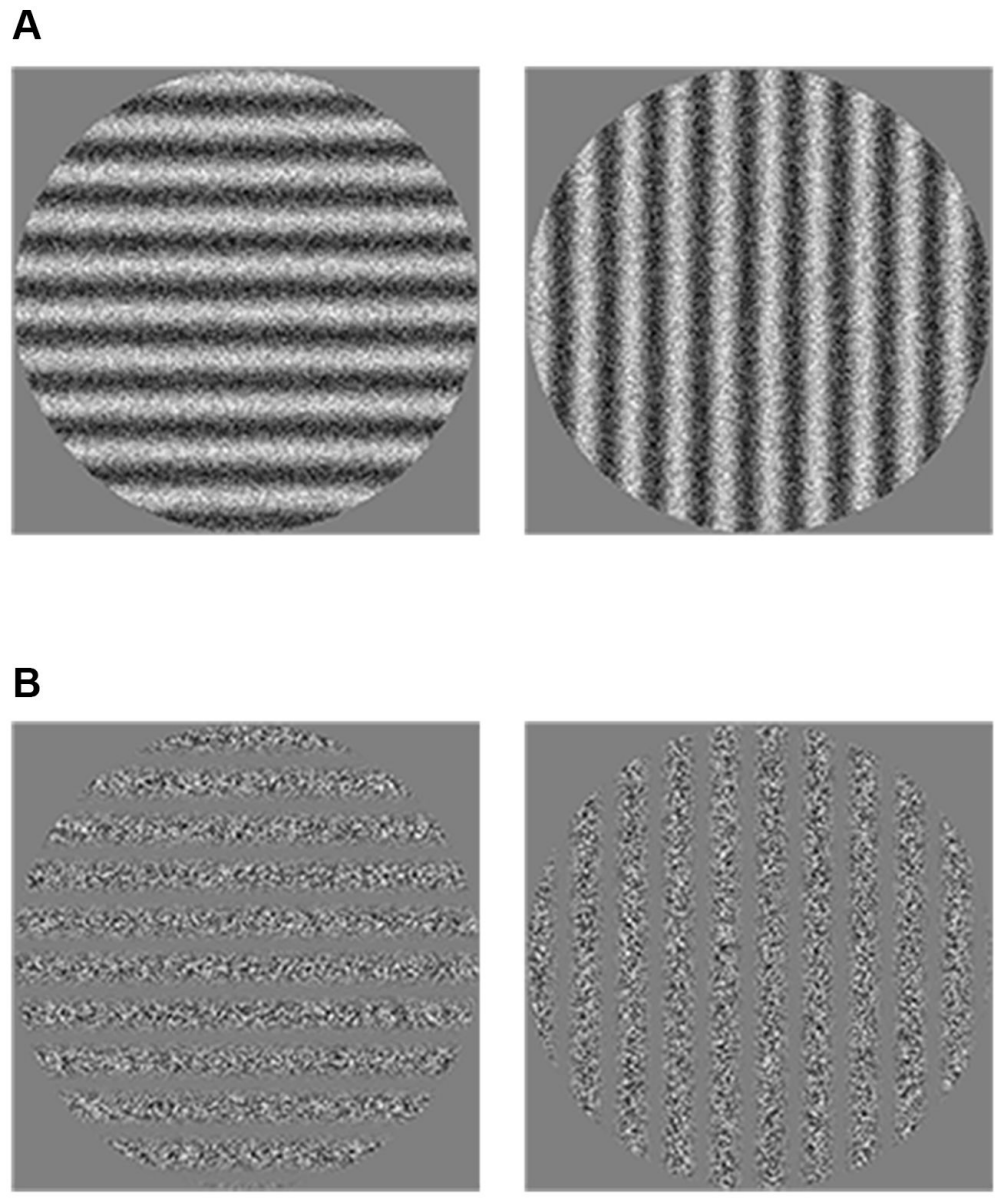
Examples of the visual stimuli, at very high contrast levels for publication purposes. A) First-order condition: Luminance-defined gratings oriented either horizontally or vertically; B) Second-order condition: Texture-defined gratings oriented either horizontally or vertically.

Visual stimuli were created based on those used by Bertone et al. [36]. They were stationary vertical or horizontal gratings of 1 cycle per degree (c/deg) presented within a circular aperture subtending 10 degrees of visual angle when viewed from a distance of 114 cm. The average luminance of the display was fixed at 30 cd/m^2^. Gratings were presented for 750 ms on a static-noise background (Michelson contrast of 50%) which was always present. During inter-stimulus intervals, a small white cross appeared at the center of the screen to announce the next trial. The orientation of the gratings was defined in terms of either luminance modulation (first-order condition: FO) or texture modulation (second-order condition: SO) (see Figure 1). FO gratings were created by adding the greyscale noise to a modulating sinewave, while their SO counterparts were constructed by multiplying these two elements. Small noise dots of 2 x 2 arc min were used to avoid the possibility of local FO artifacts in our SO stimuli, as recommended by Smith and Ledgeway [51]. Finally, to minimize the presence of non-linearities in the stimuli, the monitor was *gamma* corrected *with a photometer on a regular basis*. The modulation depth (or apparent contrast) of the gratings was manipulated by varying the amplitude of the sinewave, as defined by one of these two formulas depending on the experimental condition: 

Luminance modulation (FO) = (L_max_- L_min_) / (L_max_ + L_min_), where L_max_ and L_min_ are the maximum and minimum mean local luminance in the visual stimulus.

Contrast modulation (SO) = (C_max_- C_min_)/ (C_max_ + C_min_), where C_max_ and C_min_ are the maximum and minimum local contrasts in the visual stimulus. 

### Psychophysical Procedure

The method of constant stimuli was used to assess children’s visual sensitivity to the stimuli. Since FO and SO thresholds are on two different scales of sensitivity (i.e., FO gratings are always more salient than their SO counterparts at the same modulation depth value), they were analyzed separately. FO and SO stimuli were presented within an experimental session in a pseudo-random sequence in order to avoid adaptation and/or practice effects. Based on previous work with typically developing children [46] and on our own pilot data with three children, the following modulation depths were used for the psychophysical task in order to equate the relative saliency of the FO and SO stimuli: FO = 5%, 2%, 1%, 0.5% and 0.25% ; SO = 50%, 20%, 15%, 10% and 5%. 

The children were seated 114 cm from the monitor and had a binocular view of the screen. FO and SO stimuli were shown one at a time for 750 ms. Using a two alternative forced-choice paradigm, the children’s task was to fixate the center of the stimuli and identify the orientation of each grating ( vertical or horizontal). Depending on their verbal abilities or preference, the children could either point their answer on a response card depicting each orientation, or respond verbally; “lines standing up” or “lines lying down”. Twenty practice trials were administered to familiarize the children with the procedure before the experimental sequence. The experimental task consisted of 200 trials, comprised of 10 repetitions of the following combination; 2 conditions (FO or SO) x 5 modulation depths x 2 orientations (vertical or horizontal). Every child was observed during the psychophysical task to ensure that he/she maintained the correct viewing distance. In order to control for attentional fluctuations, each trial was initiated by an examiner seated next to the child throughout testing.

### Electrophysiological Procedure

The electrophysiological procedure was intended to compare brain activity associated with FO and SO processing. Since FO cues are generally more salient than their SO counterparts, and considering that VEPs are sensitive to the parameter of saliency [52], FO and SO gratings were equated in terms of perceived visibility based on pilot testing. Based on previous thresholds measurements in typically developing children [45] and on our own pilot data with three children, FO modulation depth was fixed at 6% while SO modulation depths was fixed at 100% for stimuli used during electrophysiological recording. These contrast stimuli were salient enough to obtain reliable VEPs for every child tested. FO (6%) and SO (100%) gratings, oriented either vertically or horizontally, were shown during 750 ms each, in two pseudo-random sequences. Each grating was presented 80 times (40 times per sequence), for a total of 320 trials. The inter-stimulus interval varied randomly between 900 and 1500 ms. Brain activity was recorded continuously during the experimental procedure with a high-density electrophysiological system of 128 electrodes, covering the whole head (HydroCel sensor net: Electrical Geodesics Inc., Eugene, OR, USA). The reference was the vertex and impedances were kept below 40kΩ [53]. EEG signals were acquired with a band-pass filter (0.1 to 100 Hz) on a G4 Macintosh computer running the Net Station program and were amplified using Net Amps 200.

Visual stimuli were viewed passively, with children fixating the center of the display (sometimes seating on their parent’s lap, as needed). A passive viewing condition was chosen in order to accommodate children with lower language-based abilities and/or greater self-control problems. In order to adequately monitor children’s behaviour, one experimenter was always present in the testing room and made sure that children maintained fixation and a viewing distance of 114 cm throughout the procedure. When children were seated on their parent’s lap, the position of the screen monitor was changed accordingly to respect the proper visual angle/viewing distance of 114 cm. By pressing a button on an electronic device, this experimenter was signalling to a second experimenter in charge of the EEG acquisition in the adjacent room whether a trial was valid or not. The EEG acquisition was paused whenever a child looked away from the screen, moved excessively or appeared distracted, tired or excited. An additional experimental sequence of 80 stimuli was administered whenever a child produced too many artifacts. This occurred for two autistic children and one typical child. 

### Psychophysical Analyses

Individual psychophysical performances were analyzed with the Matlab software (MATLAB). For each condition (FO, SO), the percentage correct at each modulation level (i.e., contrast) was fitted to a Weibull psychometric function. Orientation-identification thresholds were defined as the modulation level corresponding to 75% correct performance. Student’s *t*-tests were subsequently performed to assess whether the mean thresholds of typically developing and autistic children were significantly different from each other for each experimental condition. To assess the response consistency through the task, early and late trials (i.e., first-half versus second-half) were also compared for each FO and SO contrast and for each group (TYP, AUT).

### Electrophysiological Analyses

Brain Vision software, version 1.05 (Brain Vision Products, Munich, Germany), was used to process the EEG recording and obtain VEPs. EEG data was first filtered and digitized (bandwidth: 1-50 Hz; *24 dB*/ octave; 250 Hz sampling rate), referenced to the average potential [54] and then divided into epochs of 600 ms starting 100 ms before stimulus onset. *Eye* movement *correction* was applied using the algorithm of *Gratton, Coles* and Donchin [55] according to the activity recorded at channels 8 and 25 of the HydroCel sensor net. EEG artifacts were rejected by visual inspection and on the basis of an amplitude criterion of ± 100 μV. DC detrend and baseline correction (-100 ms to the stimulus onset) were employed on the data. 

The mean number of recorded trials during EEG acquisition (before artifact rejection) did not differ between the two groups of participants for both FO (*M*_AUT_ = 161 trials, s.d. = 18.47 ; *M*_TYP_ = 155.63 trials, s.d. = 20.92 ; *t*(32) = 0.78, *p* = 0.44) and SO conditions (*M*_AUT_ = 161.4 trials, s.d. = 19.1 ; *M*_TYP_ = 155.21 trials, s.d. = 20.54 ; *t*(32) = 0.9, *p* = 0.38). Statistical analyses on the number of clean trials that was used for averaging after artifact rejection also showed no significant effects of Group or Condition, nor was there a significant interaction between Condition and Group (all *p*s > 0.22). The mean number of clean trials was 142.13 and 141.93 for FO and SO stimuli, respectively, in the control group, and 150.98 and 152.16 for FO and SO stimuli, respectively, in the autism group. 

Visual inspection of the grand-average VEP waveforms of both groups of children revealed highly similar spatiotemporal peaks and troughs. Three temporal windows of cerebral activity were identified at approximately 140 ms (P140 component), 230 ms (N230 component) and 340 ms (P340 component) after stimulation onset. Channels of interest were selected by visual inspection of the grand-average maps and included occipital electrodes (E69, E70, E74, E75, E82, E83), as well as occipito-temporal and parietal electrodes over the left (E51, E52, E53, E57, E58, E59, E64, E65) and right hemispheres (E78, E79, E84, E85, E86, E87, E90, E92, E95, E96, E100).

VEPs in response to vertical and horizontal gratings were visually inspected and the orientation did not appear to modulate VEP waveforms, which was confirmed by BrainVision assisted *t*-tests. EEG segments were therefore collapsed across grating orientation. All averaged VEP waveforms were subjected to a reference-free scalp current source density (CSD) analysis using the spherical spline interpolation of the surface voltage recordings [56,57]. CSD distributions are obtained by computing second spatial derivatives (the Laplacian) of the scalp field potentials and show the scalp areas where the radial (transcranial) currents flowed into (‘’sinks’’) or out of the brain (‘’sources’’) [54]. By comparison to VEPs, CSD estimates are independent of the reference electrode, sharpen the *spatial resolution* of recorded data, and act as spatial filters that amplify the contribution of local cortical generators and diminish the contribution of distant or subcortical generators. They thus provide superior resolution of superficial neuronal events. Mathematical transformations were performed using a Matlab toolbox [58], with the following computation parameters: 50 iterations; spline interpolation constant m = 4; λ smoothing constant = 1.0e-6. 

**Figure 2:**
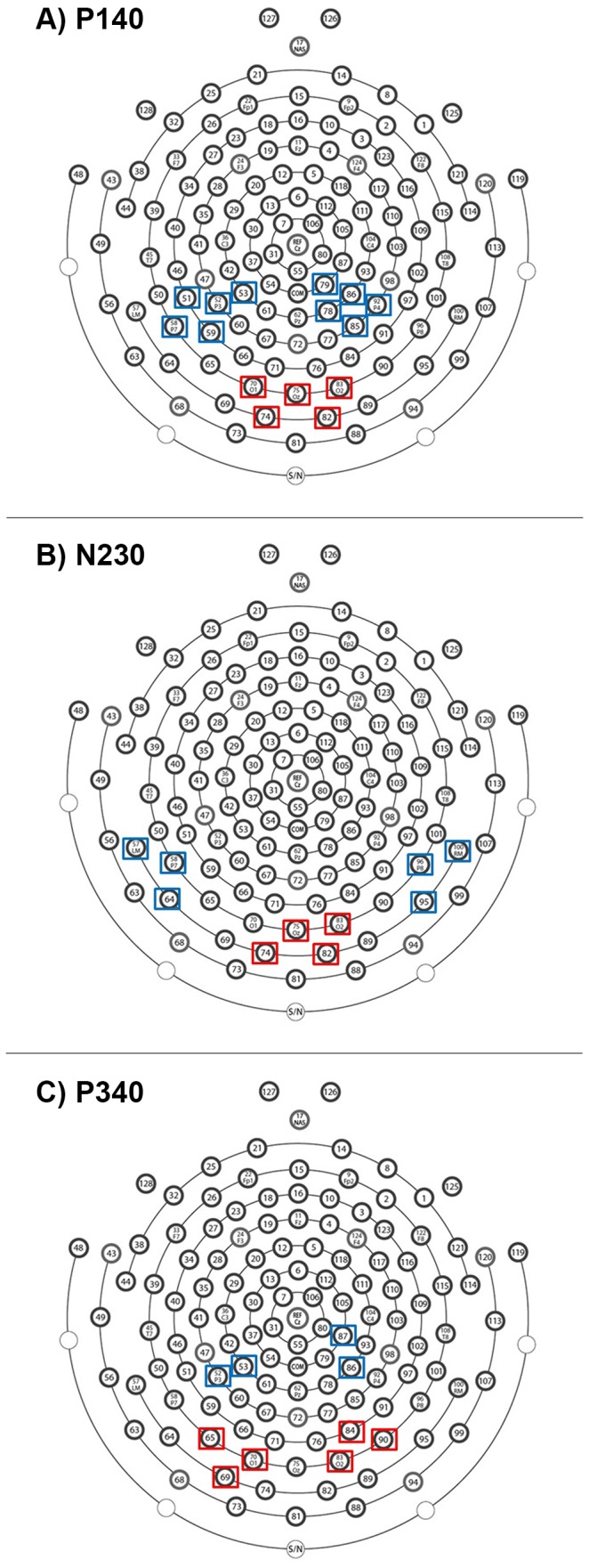
Sensor layout of the Hydrocel Geodesic Sensor Net - 128 channels. Electrodes used to carry out the statistical analyses on CSD estimates are surrounded by a square, separately for each component of interest: A) P140, B) N230, and C) P340. Red squares represent the electrodes used to compute source activity, while blue squares represent the electrodes used to compute sink activity.

CSD waveforms and topographic maps were highly similar in typical and autistic children at all temporal windows of interest. Based on visual inspection of the grand-average maps, three or four electrode clusters were computed by averaging the responses at electrodes covering sink and source activity, separately for each component (P140, N230, P340) (see Figure 2). Amplitudes were estimated as the mean value, expressed in units of µV/cm^2^, averaged across electrodes of interest within specified time-windows (P140: 124 to 172 ms; N230: 212 to 260 ms; P340: 324 to 372 ms). Latencies were measured to the time from stimulus onset to the most positive or negative amplitude value (peak latencies). 

CSD estimates were analyzed using repeated measures analysis of variance (ANOVA) with Group as the between-subjects factor (TYP, AUT), Condition as a within-subject factor (FO, SO) and, when appropriate, Hemisphere as a second within-subjects factor (Left, Right). The Hemisphere factor was not included for the P140 and N230 source analyses (i.e., sources were located on the medial side of the occipital area). Post hoc analyses were conducted using paired t-tests with Bonferroni corrected *p* values. For the P340 amplitude analyses only, non-parametric Wilcoxon tests for paired samples were applied to test the differences between FO and SO *source-sink* dynamics, as the data were not normally distributed in the autism group. For transparency, marginally significant effects (0.05 < *p* < 0.09) are also reported. Outlier scores (z-scores of + 3.0) were replaced with values corresponding to z-scores of + 2.0. This procedure resulted in changing two CSD values in the typical group and two CSD values in the autism group. For all ANOVA significant effects, effect size was calculated using partial eta-squared (ηρ^2^). 

## Results

### Psychophysical Results

**Figure 3:**
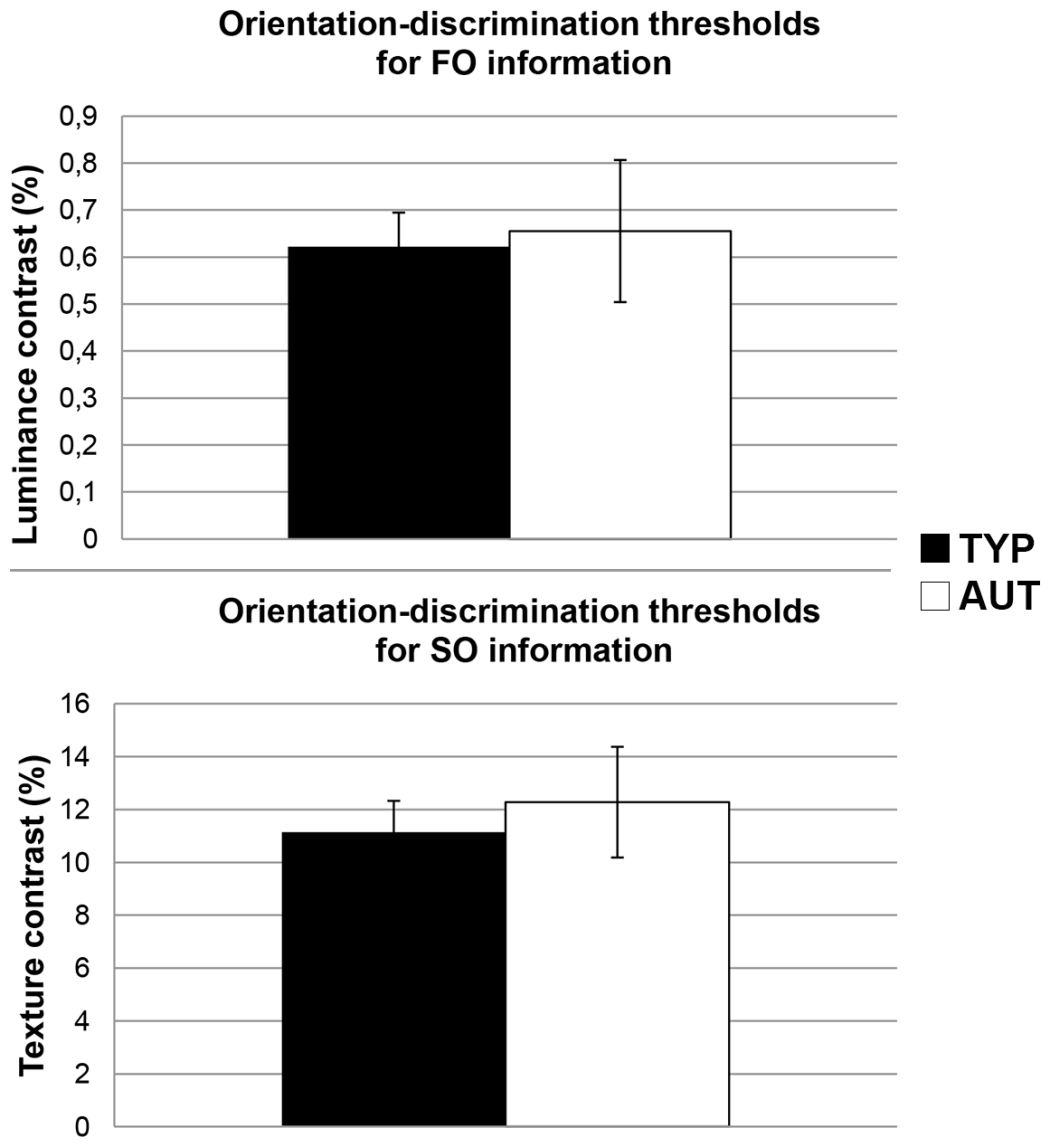
Behavioural results.

As illustrated in Figure 3, FO thresholds did not differ between autistic children and their typically developing peers (*M*_AUT_ = 0.66%, s.d. = 0.55; *M*_TYP_ = 0.62%, s.d. = 0.33; *t*(32) = 0.22, *p* = 0.83), nor did their SO thresholds (*M*_AUT_ = 12.28%, s.d. = 7.55; *M*_TYP_ = 11.14%, s.d. = 5.43 ; *t*(32) = 0.51, *p* = 0.61). These results are in contrast with those previously obtained with autistic adults [36]. In order to rule out the possibility of fatigue or learning effects, we compared performance of both groups obtained in the first 100 trials and in the remaining 100 trials. For both groups, the analyses revealed no performance discrepancy for any of the FO or SO contrast (*p*s > 0.1 at all paired *t*-tests). 

Orientation-discrimination thresholds for typically developing children (TYP: black) and autistic children (AUT: white). Error bars represent standard error of means. 

### Current Source Density (CSD)

Mean amplitude and peak latency values of all three CSD components (P140, N230, P340) are listed in Table 2 and Table 3, respectively, separated by participant group (TYP, AUT) and experimental condition (FO, SO). 

**Table 2:** Mean amplitude values of CSD responses (in µV/cm^2^ + *SD*) to luminance-defined (first-order: FO) and texture-defined (second-order: SO) visual stimuli in typically developing and autistic children.

			**Typical Group**	**Autism Group**
**P140**	**Occipital Source**	**FO**	1.18 + 0.6	0.94 + 0.44
		**So**	0.94 + 0.05	0.77 + 0.43
	**Left Parietal Sink**	**FO**	-0.36 + 0.15	-0.3 + 0.16
		**So**	-0.29 + 0.13	-0.26 + 0.12
	**Right Parietal Sink**	**FO**	-0.27 + 0.17	-0.26 + 0.19
		**So**	-0.22 + 0.16	-0.22 + 0.21
**N230**	**Left Temporal Sink**	**FO**	-0.27 + 0.36	-0.19 + 0.2
		**So**	-0.39 + 0.41	-0.22 + 0.23
	**Right Temporal Sink**	**FO**	-0.33 + 0.21	-0.25 + 0.28
		**So**	-0.5 + 0.29	-0.37 + 0.31
	**Occipital Source**	**FO**	0.34 + 0.49	0.32 + 0.34
		**So**	0.49 + 0.59	0.28 + 0.32
**P340**	**Left Parietal Sink**	**FO**	-0.12 + 0.14	-0.1 + 0.13
		**So**	-0.16 + 0.17	-0.14 + 0.2
	**Right Parietal Sink**	**FO**	-0.1 + 0.08	-0.08 + 0.08
		**So**	-0.14 + 0.09	-0.11 + 0.11
	**Left Occipito-Temporal Source**	**FO**	0.29 + 0.18	0.18 + 0.2
		**So**	0.35 + 0.16	0.2 + 0.18
	**Right Occipito-Temporal Source**	**FO**	0.26 + 0. 24	0.22 + 0.17
		**So**	0.33 + 0.24	%1.1 + 0.23

**Table 3:** Mean latency values of CSD responses (in milliseconds + *SD*) to luminance-defined (first-order: FO) and texture-defined (second-order: SO) visual stimuli in typically developing and autistic children.

			**Typical Group**	**Autism Group**
**P140**	**Occipital Source**	**FO**	138.74 + 15.61	138.93 + 17.07
		**So**	134.74 + 18.38	137.07 + 19.45
	**Left Parietal Sink**	**FO**	143.58 + 18.13	145.07 + 20.07
		**So**	144.21 + 20.2	142.93 + 22.5
	**Right Parietal Sink**	**FO**	135.16 + 13.5	143.2 + 17.45
		**So**	132.63 + 12.53	142.67 + 18.8
**N230**	**Left Temporal Sink**	**FO**	226.74 + 29.4	238.13 + 30.46
		**So**	234.95 + 20.78	242.13 + 24.61
	**Right Temporal Sink**	**FO**	217.89 + 17.81	235.20 + 19.95
		**So**	224.63 + 19.24	237.07 + 23.54
	**Occipital Source**	**FO**	224.21 + 28.89	228.0 + 27.34
		**So**	224.84 + 21.77	232.53 + 24.79
**P340**	**Left Parietal Sink**	**FO**	338.95 + 26.79	338.67 + 27.33
		**So**	342.11 + 29.41	347.2 + 21.39
	**Right Parietal Sink**	**FO**	338.95 + 28.65	340.0 + 22.68
		**So**	340.42 + 24.98	344.27 + 22.09
	**Left Occipito-Temporal Source**	**FO**	336.63 + 21.59	337.87 + 32.03
		**So**	333.68 + 22.12	342.13 + 21.64
	**Right Occipito-Temporal Source**	**FO**	335.37 + 32.72	329.87 + 24.65
		**So**	341.89 + 28.98	326.13 + 22.01

#### P140 component

**Figure 4:**
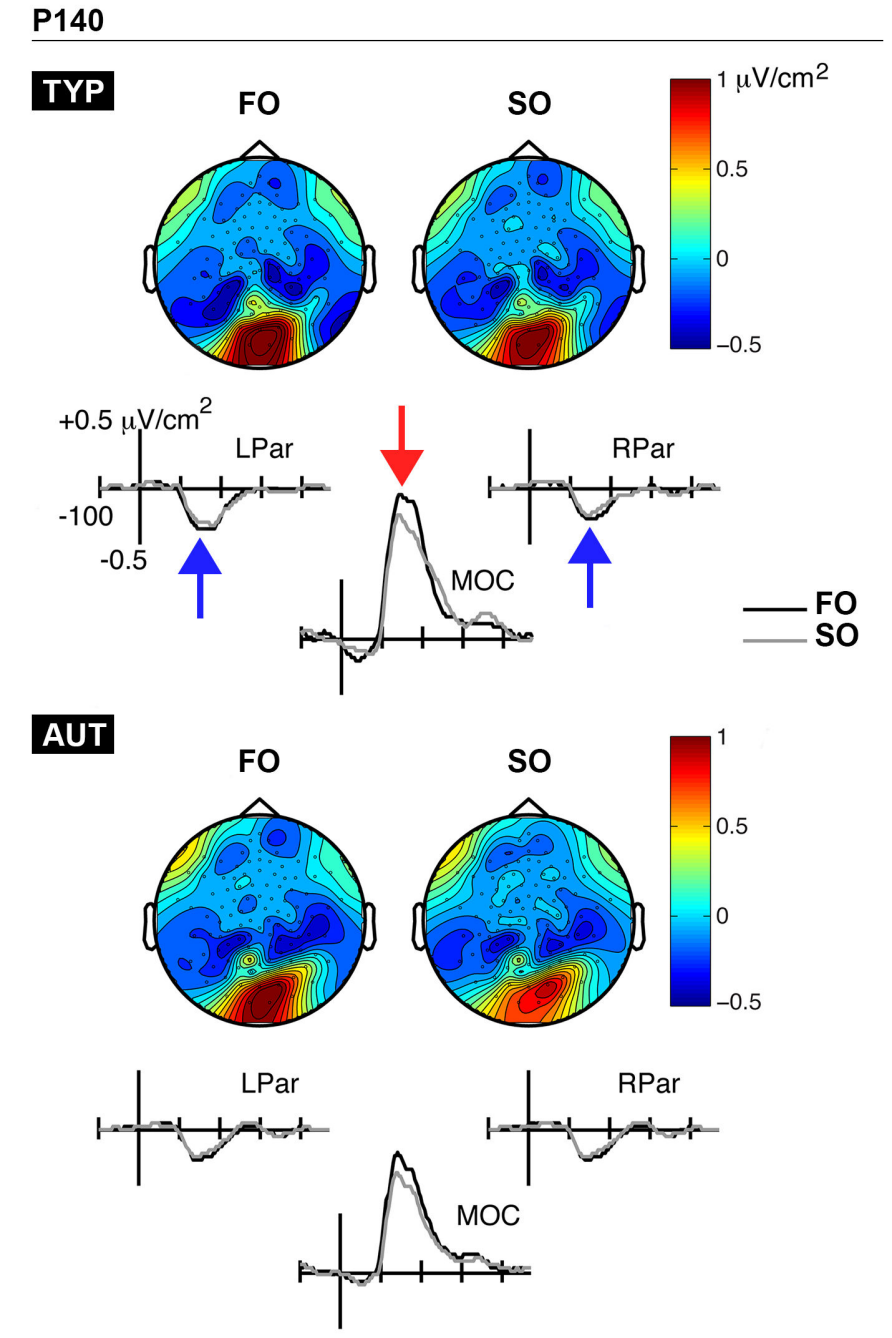
P140 time-window. CSD waveforms and topographic maps associated with first-order (FO) and second-order (SO) conditions are presented separately for typically developing (TYP; top panel) and autistic children (AUT; bottom panel). FO and SO conditions are represented with black and gray waveforms, respectively. Source activity is identified with red arrows, while sink activity is depicted by blue arrows. MOC, midline occipital; LPar, left parietal; RPar, right parietal.

Figure 4 displays the grand-average CSD waveforms and topographic maps for the P140 time-window, separately by group (TYP, AUT). The maps indicated the presence of bilateral sinks over parietal regions (left: E51, E52, E53, E58, E59; right: E78, E79, E85, E86, E92), along with a more pronounced source that was distributed over the midline occipital region (E70, E74, E75, E82, E83). Two-way repeated measures ANOVAs (Group x Condition) were carried out on source measurements, while three-way repeated measures ANOVAs (Group x Condition x Hemisphere) were carried out on sink measurements.

Regarding latency analyses, the P140 source peaked at similar time points to both FO and SO gratings (*F*(1,32) = 1.18, *p* = 0.29; *M*_FO_ = 139 ms; *M*_SO_ = 136 ms). The Group factor and all interactions involving Group factor were not significant (all *p*s > 0.7). The P140 sinks also peaked at similar time points to both FO and SO gratings (*F*(1,32) = 0.2, *p* = 0.66; *M*_FO_ = 142 ms; *M*_SO_ = 141 ms). There was a significant effect of Hemisphere (*F*(1,32) = 4.65, *p* < 0.05, ηρ^2^ = 0.13) and a marginally significant Group x Hemisphere interaction (*F*(1,32) = 3.03, *p* = 0.09, ηρ^2^ = 0.09). More specifically, the P140 sink reached its maximal amplitude earlier over the right hemisphere (*M* = 138 ms), as compared to the left (*M* = 144 ms), and this effect tended to be stronger in typical children (*M*_left_ = 144 ms; *M*_right_ = 134 ms) as compared to autistics (*M*_left_ = 144 ms; *M*_right_ = 143 ms).

Regarding amplitude analyses, both P140 source (*F*(1,32) = 38.17, *p* < 0.001, ηρ^2^ = 0.54) and sinks (*F*(1,32) = 34.8, *p* < 0.001, ηρ^2^ = 0.52) were significantly modulated by the experimental conditions. More specifically, the set of source and sinks was clearly more active for FO (*M*_source_ = 1.06 µV/cm^2^; *M*_sinks_ = -0.3 µV/cm^2^) than SO stimuli (*M*_source_ = 0.85 µV/cm^2^; *M*_sinks_ = -0.25 µV/cm^2^). The P140 sinks and source were comparable in both groups of children and all comparisons involving the Group factor did not yield significant differences (all *p*s > 0.19). With respect to the Hemisphere factor, there was a marginally significant effect of Hemisphere for the P140 sinks (*F*(1,32) = 3.63, *p* = 0.07, ηρ^2^ = 0.1). This suggested slightly greater amplitudes over the left hemisphere (*M* = -0.3 µV/cm^2^), as compared to the right (*M* = -0.24 µV/cm^2^). 

#### N230 component

**Figure 5:**
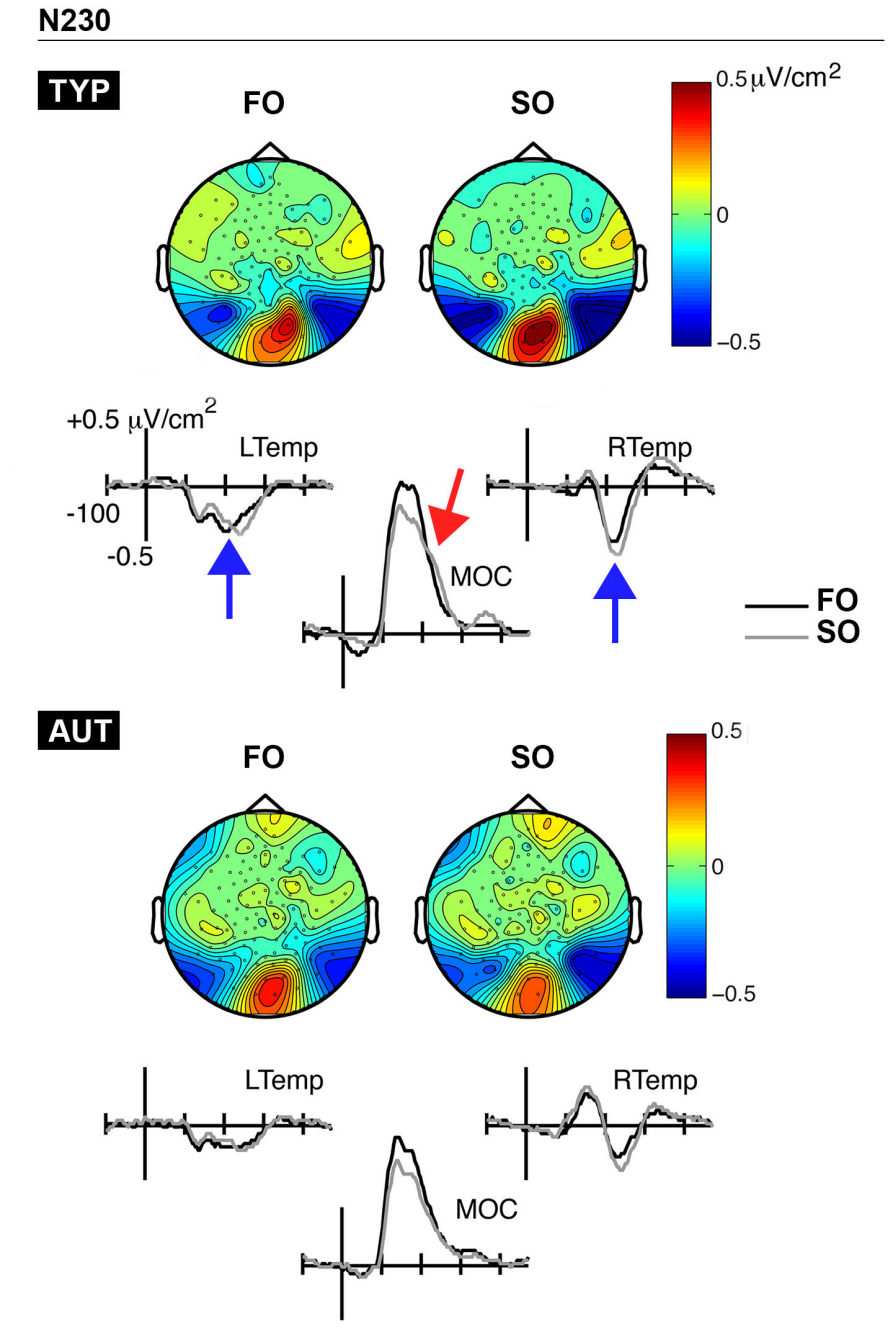
N230 time-window. CSD waveforms and topographic maps associated with first-order (FO) and second-order (SO) conditions are presented separately for typically developing (TYP; top panel) and autistic children (AUT; bottom panel). FO and SO conditions are represented with black and gray waveforms, respectively. Source activity is identified with red arrows, while sink activity is depicted by blue arrows. MOC, midline occipital; LTemp, left temporal; RTemp, right temporal.

Figure 5 displays the grand-average CSD waveforms and topographic maps for the N230 time-window, separately by group (TYP, AUT). Maps showed bilateral sinks over temporal regions (left: E57, E58, E64; right: E95, E96, E100), with a concurrent but reduced source over the midline occipital region (E74, E75, E82, E83). Two-way repeated measures ANOVAs (Group x Condition) were carried out on source measurements, while three-way repeated measures ANOVAs (Group x Condition x Hemisphere) were carried out on sink measurements.

With respect to latency analyses, the N230 source latencies were comparable between the two conditions (*F*(1,32) = 0.34, *p* = 0.57; *M*_FO_ = 226 ms; *M*_SO_ = 229 ms) and across groups (*F*(1,32) = 0.55, *p* = 0.46; *M*_TYP_ = 225 ms; *M*_AUT_ = 230 ms). For the N230 sinks, there was a marginally significant effect of Condition (*F*(1,32) = 3.76, *p* = 0.06, ηρ^2^ = 0.11; *M*_FO_ = 229 ms; *M*_SO_ = 235 ms), a marginally significant effect of Hemisphere (*F*(1,32) = 3.36, *p* = 0.08, ηρ^2^ = 0.1; *M*_left_ = 235 ms; *M*_right_ = 229 ms), and a marginally significant effect of Group (*F*(1,32) = 3.56, *p* = 0.07, ηρ^2^ = 0.1; *M*_TYP_ = 226 ms; *M*_AUT_ = 238 ms).

With respect to amplitude analyses, the pattern of activity at both N230 source (*F*(1,32) = 9.71, *p* < 0.01, ηρ^2^ = 0.23) and sinks (*F*(1,32) = 3.8, *p* = 0.06, ηρ^2^ = 0.11) differed or tended to differ between groups. In typical children, SO stimuli elicited markedly more prominent amplitude values than FO stimuli at the source (*t*(18) = -3.76, *p* < 0.01; *M*_FO_ = 0.34 µV/cm^2^; *M*_SO_ = 0.49 µV/cm^2^) and sinks (*t*(18) = 5.18, *p* < 0.001; *M*_FO_ = -0.3 µV/cm^2^; *M*_SO_ = -0.45 µV/cm^2^). In autistics, the FO-SO difference was non-significant at the source (*t*(14) = 0.88, *p* = 0.4), while it was smaller over the sinks (*t*(14) = 2.57, *p* < 0.05; *M*_FO_ = -0.22 µV/cm^2^; *M*_SO_ = -0.29 µV/cm^2^). Notably, for both groups, the Condition effect on sink activity was greater in the right than in the left hemisphere, as indicated by a significant Condition x Hemisphere interaction (*F*(1,32) = 7.2, *p* < 0.05, ηρ^2^ = 0.18). 

#### P340 component

**Figure 6:**
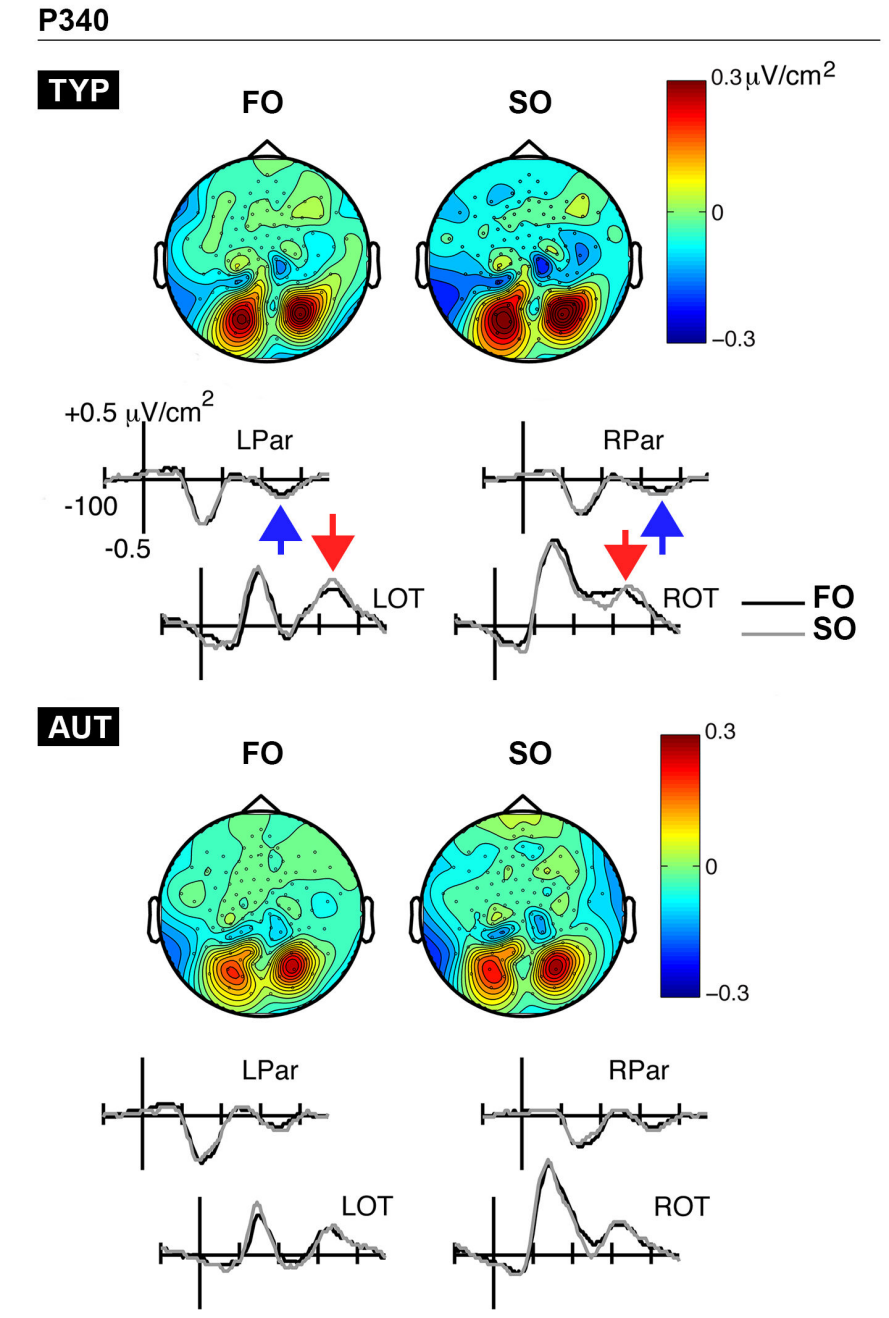
P340 time-window. CSD waveforms and topographic maps associated with first-order (FO) and second-order (SO) conditions are presented separately for typically developing (TYP; top panel) and autistic children (AUT; bottom panel). FO and SO conditions are represented with black and gray waveforms, respectively. Source activity is identified with red arrows, while sink activity is depicted by blue arrows. LPar, left parietal; RPar, right parietal; LOT, left occipito-temporal; ROT, right occipito-temporal.

Figure 6 displays the grand-average CSD waveforms and topographic maps for the P340 time-window, separately by group (TYP, AUT). Simultaneous bilateral pairs of sinks (left: E52, E53; right: E86, E87) and sources (left: E65, E69, E70; right: E83, E84, E90) were elicited over the parietal and occipito-temporal sites, respectively. Three-way repeated measures ANOVAs (Group x Condition x Hemisphere) were carried out on latency measurements, separately for source and sink activity. As mentioned above, amplitude measurements were analyzed using non-parametric Wilcoxon tests for paired samples because the assumption of normality was violated. Notably, the Hemisphere factor was not included in these analyses because there is no non-parametric counterpart for factorial repeated measures designs.

Regarding latency analyses, there was a marginally significant interaction between Group and Hemisphere for P340 sources (*F*(1,32) = 3.64, *p* = 0.07, ηρ^2^ = 0.1). More specifically, the autism group tended to show longer latencies over the left hemisphere (*M* = 340 ms) as compared to the right (*M* = 328 ms), *F*(1,14) = 10.59, *p* < 0.05. In contrast, typical children showed more similar P340 sources latencies over both cerebral hemispheres (*F*(1,18) = 0.28, *p* = 0.6; *M*_left_ = 335 ms; *M*_right_ = 339 ms). With respect to P340 sinks, the latencies were comparable between the two conditions (*F*(1,32) = 1.94, *p* = 0.17; *M*_FO_ = 339 ms; *M*_SO_ = 343 ms) and across groups (*F*(1,32) = 0.12, *p* = 0.74; *M*_TYP_ = 340 ms; *M*_AUT_ = 343 ms). All interactions involving Group or Hemisphere factors were also not significant (all *p*s > 0.51).

Regarding amplitude analyses, the two groups showed different sink-source patterns in relation to experimental conditions. In typical children, sink and source activity was significantly or marginally significantly higher for SO than FO stimuli over the left (*Z* = -1.77, *p* = 0.08, *r* = -0.41; *Mdn*_FO_ = -0.13 µV/cm^2^; *Mdn*_SO_ = -0.14 µV/cm^2^) and right parietal regions (*Z* = -2.62, *p* < 0.01, *r* = -0.6; *Mdn*_FO_ = -0.09 µV/cm^2^; *Mdn*_SO_ = -0.14 µV/cm^2^), as well as over the right occipito-temporal region (*Z* = -2.13, *p* < 0.05, *r* = -0.49; *Mdn*_FO_ = 0.26 µV/cm^2^; *Mdn*_SO_ = 0.3 µV/cm^2^). In contrast, autistic children did not show any differences in sink or source activity between FO and SO conditions over the left parietal (*Z* = -1.08, *p* = 0.28), right parietal (*Z* = -1.59, *p* = 0.11), left occipito-temporal (*Z* = -1.25, *p* = 0.21), and right occipito-temporal regions (*Z* = 0.0, *p* = 1.0).

### Statistical Control for Medication

In order to eliminate any potential bias or influence medication could have had on brain activity, we repeated all *t*-tests, ANOVAs, and non-parametric Wilcoxon tests stated above after removing the children who were on medication. This resulted in the removal of six children. The two groups remained matched on chronological age and fluid nonverbal intellectual ability (all *p*s > 0.1). The exclusion of these six children did not alter the psychophysical or electrophysiological findings, with two exceptions. More specifically, the marginally significant Group x Hemisphere interaction related to the P140 sink latencies became non-significant, as well as the marginally significant effect of Condition related to the N230 sinks latencies. Overall, these test results indicate that the main findings with respect to Group x Condition interactions were not driven by children who were on medication at the time of the study.

### Relationships between Behavioural and Electrophysiological Measures

We further examined how well behavioural data (FO-SO thresholds) were correlated with the electrophysiological measures. In the control group, FO thresholds only correlated with P140 source activity (r = -0.48, *p* < 0.05). This indicated that typical children who were better at discriminating the orientation of luminance-defined gratings also showed greater brain activity over the occipital region at around 140 ms. In the control group, there was no correlation between SO thresholds and any of the electrophysiological measures.

In the autism group, FO (r = 0.6, *p* = 0.05) and SO thresholds (r = 0.61, *p* < 0.05) only correlated (or tended to correlate) with the P140 sink activity over the left hemisphere. Therefore, autistic children who were better at discriminating the orientation of luminance- and texture-defined gratings showed greater (more negative) brain activity over the left parietal region at around 140 ms.

Behavioural data did not correlate with any latency measures of brain activity in either typical (all *p*s > 0.1) or autistic children (all *p*s > 0.12). 

### Exploratory Analyses

On an exploratory basis, we further investigated the effect of chronological age and cognitive skills on our test measures. We first examined whether the psychophysical thresholds correlated with chronological age and RPM or PPVT scores by calculating Pearson correlation coefficients. With respect to electrophysiology, the exploratory analyses were limited to the most relevant findings in our data, namely the difference in brain activity between FO and SO trials (as shown by CSD amplitude values). Latency variables were not included, as they failed to reveal significant differences across groups, or significant interactions between Group and Condition factors. As such, for each component (P140, N230, P340), we computed new variables that were the difference in amplitude between FO and SO conditions. Notably, greater FO-SO difference in brain activity indicated better visual differentiation between the two classes of stimuli, which was thought to reflect more mature neurovisual processes. 

Due to the exploratory nature of these correlations, we performed unadjusted tests at the 0.05 level of significance. Hence, significant test results are viewed as providing preliminary information on relationships in the data and should be subject to more rigorous examination in future investigations. 

#### Relationships with age

When the data from typical and autistic children were analyzed together, FO and SO psychophysical measures negatively correlated with chronological age (FO: r = -0.35, *p* < 0.05; SO: r = -0.36, *p* < 0.05). In line with the literature [45,46], this suggests that both visual systems are developing between the ages of 6 and 11 years-old. However, when groups were analyzed separately, the only correlation that remained significant was the relationship between chronological age and SO threshold in autistic children (r = -0.6, *p* < 0.05). In our opinion, statistical power was deemed to be insufficient to reveal significant relationships when data was analyzed separately by group. 

Correlations between chronological age and amplitude differences between FO and SO conditions were all non-significant for both the control (all *p*s > 0.29) and autistic group (all *p*s > 0.11).

#### Relationships with cognitive skills

In the control group, the correlation between SO thresholds and RPM scores approached significance (r = -0.39, *p* = 0.08). In addition, *there was a marginally significant correlation between RPM scores* and N230 source activity (r = 0.44, *p* = 0.06). These results suggest that typical children with higher nonverbal reasoning skills tended to show enhanced sensitivity towards SO visual attributes, as well as greater FO-SO differentiation in brain activity over the occipital cortex at around 230 ms. Correlations between our psychophysical/electrophysiological measures and PPVT scores were all non-significant in typical participants (*all* p*s > 0.1*)*.*

In the autism group, no significant relationship was found between psychophysical/electrophysiological measures and RPM scores (*all* p*s > 0.17*)*. On the other hand, there was a significant correlation between PPVT scores and P340 sink activity* over the left hemisphere (r = -0.52, *p* < 0.05). This indicated that autistic children with higher verbal aptitudes showed greater FO-SO differentiation in brain activity over the left parietal cortex at around 340 ms.

## Discussion

We have investigated whether the dissociation between luminance-defined (FO) and texture-defined (SO) vision in autistic adults is present as early as 6 to 11 years of age. We measured thresholds using the method of constant stimuli and recorded the brain responses to luminance-defined and texture-defined patterns. Based on the complexity-specific hypothesis, we expected to find in autistic children superior performance and enhanced/faster cerebral response for the FO condition, concomitant with inferior performance and reduced/delayed cerebral response for the SO condition. This series of experiments was also devised to document the cortical representation of these two systems in typically developing children. This should provide a comparison basis to reveal alterations, if any, of the cortical representation of these systems in autism. 

### Cortical Representation of Luminance- and Texture-Defined Information Processing in Typically Developing Children

To our knowledge, there are no electrophysiological investigations of texture-defined static attributes in typical children outside the field of texture segregation [59]. In our control group, brain activity was modulated as a function of visual attribute at all three time-windows (P140, N230, P340). This is consistent with the notion that FO and SO cerebral networks overlap, but appear to be differentially recruited over time. At about 140 ms, brain activity predominantly showed a mesial positive source localized at occipital sites that was found to be larger in amplitude for luminance as compared to texture gratings. Given the fact that CSD analyses sharpen EEG topographies and yield measures that more closely represent underlying cortical generators, the primary visual cortex V1 appeared to contribute to the P140 signal, likely in collaboration with extrastriate visual areas. Furthermore, we believe our P140 component primarily reflects the arrival time of the afferent visual pathway to striate and extrastriate areas, as suggested by the literature on the P1 VEP component [53,60,61]. Overall, our P140 findings are in agreement with the idea that FO information is processed earlier than SO information along the visual hierarchy and relies on striate mechanisms [23,24]. Additionally, the fact that FO (but not SO) psychophysical thresholds correlate with the brain activity within the 124-172 ms time-window strengthens the idea that the P140 component is predominantly an index of FO visual processing. 

Conversely, typical children showed greater brain activity in response to texture as compared to luminance gratings during the N230 and P340 time ranges. Critically, the N230 and P340 activity was more pronounced at occipito-lateral and parietal electrodes, which indicates a greater involvement of extrastriate visual areas in SO processing. *In contrast to FO data, there was no significant correlation between SO psychophysical thresholds and any measures of brain activity, perhaps because texture information processing is more distributed across various brain structures, or due to limited power of our analyses.*

*Overall*, the present electrophysiological results in typical children support the idea of a functional dissociation between luminance and texture visual processes, which is evident as early as 6-11 years of age. Our data are *consistent with the notion that* extrastriate areas such as V2, V3 and/or V4 are solicited to a greater extent during SO information processing [23,24,27,33]. A previous study conducted with typical adults and using stimuli similar to ours also reported two VEP components indexing SO information processing at around 200 and 336 ms [28]. In line with behavioural studies [44,45], the slight differences in latency and scalp distribution between our and Calvert and colleagues’ findings may suggest that texture pattern processing mechanisms are not fully mature in 6-to-11-years-olds, with less specific or specialized neural generators. However, non-cerebral factors such as the thickness of the skin and skull or head geometry cannot be ruled out.

### Luminance-Defined Information Processing in Autistic Children

Although previous results have demonstrated superior sensitivity to luminance-defined static gratings in autistic adults [36], we did not find lower psychophysical thresholds or enhanced/faster brain responses to luminance stimuli in autistic children. They showed similar FO thresholds to those found in their typically developing peers. There were also no absolute group differences in brain activity more closely associated with FO processing (P140). Indeed, the location of the P140 source and sinks appeared quite similar across groups. Furthermore, both typical and autistic children showed increased brain activity in response to luminance as compared to texture gratings during this early time-window (124-172 ms). 

### Texture-Defined Information Processing in Autistic Children

Behaviourally, autistic children did not demonstrate higher orientation-identification thresholds for texture gratings. With respect to electrophysiology, the location of the N230 and P340 current foci appeared to be fairly identical across groups, suggesting that similar brain structures were recruited during these two time-windows more closely associated with texture processing. However, at about 230 and 340 ms, autistics’ brain activity was not reliably enhanced for texture stimuli than luminance stimuli. This pattern of results could imply that extrastriate visual areas were not as sensitive to visual texture information in autism when compared to typical children. The complexity-specific hypothesis proposes that abnormal lateral and/or feedback connectivity within low-level visual areas explains the FO-SO behavioural dissociation [36]. We did not specifically test this assumption, as our paradigm did not allow us to isolate signals related to feedforward and/or lateral processing from feedback activity. Notably, both lateral and feedback connections are likely involved in N230 and P340 activity given the rapidity at which temporal and parietal cortices can be activated during visual perception [62]. Our results are therefore in keeping with other accounts of decreased differentiation in brain activity during the processing of visual characteristics such as spatial frequency [62-65]. Furthermore, FO and SO psychophysical thresholds were correlated with brain activity over the left parietal region at around 140 ms in the autistic group only. This finding, although preliminary, further reinforces the idea that autistics tend to process visual information defined by different physical attributes with more similar cerebral mechanisms. The atypical extraction of visual features (including but not necessarily limited to enhanced perception) thus appears to characterize early visual perception in autism for both children and adults (see also Kéïta et al., 2011 [43]). 

The findings of the current study do not support selective dorsal stream impairment in autism [15], as we found abnormal cortical responses to stationary complex gratings which predominantly stimulate the visual ventral stream. However, as recently evidenced by the study of Greenaway, Davis and Plaisted-Grant [66], and given the fact that magnocellular activity reaches V1 before parvocellular activity [62], we cannot rule out the presence of subcortical magnocellular abnormalities (e.g., lateral geniculate nucleus) that would diversely affect both ventral and dorsal visual functions. On the other hand, the present findings seem consistent with those of Vandenbroucke and colleagues [67] who also reported atypical brain activation associated with texture-defined boundaries in a group of adults with ASD. The group differences observed by Vandenbroucke et al. were however present as early as 120 ms after stimulus presentation, and likely reflected dysfunctional lateral connectivity within early visual areas. This is considerably earlier than the VEP alterations that were evidenced by the present study. Differences in visual stimuli, methodological design, and/or participant age and cognitive status could account for the discrepancy across studies. Specifically, it is not clear whether significant developmental changes exist with regard to mechanisms mediating texture information processing in individuals with ASD. A texture segregation experiment with older autistic children/teenagers (mean age of 13.3 years) indicated slightly aberrant, albeit non-significantly, visual recurrent mechanisms between extrastriate areas and V1 [43]. Overall, VEP results in autistic children (present study), teens [43], and adults [67] all suggest abnormal texture processing in autism. Different neural processes, which are not mutually exclusive, appear to contribute to this phenomenon, possibly to a different extent across period of development. 

### Developmental Trajectory of Visual Functions in Autism

The developmental trajectory of FO and SO visual functions in autism may also explain the discrepancy between children’s and adults’ findings. During typical development, mechanisms mediating FO vision undergo longer maturation during childhood than those mediating SO attributes [44,45], as typically developing children reach adult-like thresholds for static SO information before FO (i.e., 5-8 years-old versus 9-10 years-old). Given that our participants were between the ages of 6 and 11 years old, FO visual mechanisms were perhaps not fully mature and group differences could not yet be detected. The fact that both FO and SO psychophysical thresholds negatively correlated with chronological age in our data is in agreement with that hypothesis. However, one major counterargument comes from a recent study from our laboratory (Meilleur et al., submitted) that failed to replicate the FO superiority in a large group of autistic adults. Due to conflicting evidence, it thus remains unclear whether autistic adults show intact or superior processing abilities of static luminance-defined information. More research is therefore needed to understand the factors that explain the divergent findings. In the event that superior FO processing is found to be a robust visual characteristic of AS adults, our study strongly suggests that it likely emerges during adolescence. 

In contrast, our electrophysiological results are consistent with the complexity-specific hypothesis in that neuro-integrative mechanisms mediating SO processing appear to be suboptimal as early as the school-years period in autism. In that context, perhaps autistic children rely more on basic pattern processing mechanisms during visual perception which would lead, in turn, to over-developed FO mechanisms by the end of childhood. Longitudinal research would permit an examination of this hypothesis. Surprisingly, there was no relationship between children’s chronological age and the electrophysiological measures contrasting FO and SO visual processing. However, given our relatively small sample size, the correlation estimates were likely very noisy.

### Study Limitations and Future Directions

One strength of our study design was the combined psychophysical and EEG approach, as these two complementary techniques reflect different perceptual aspects [68]. However, there are also certain limits that need to be considered when interpreting the results.

Firstly, the lack of hypothesized behavioural group-differences in our study (for both luminance- and texture-defined conditions) may be due to our techniques not being optimal for our younger participants, particularly those in the autism group. Although psychophysical estimates have been obtained with typical children as young as 5 years-old with stimuli and paradigms very similar to ours [44,45,69], such paradigms may not be as effective for obtaining reliable thresholds for children with neurodevelopmental conditions such as autism. This is reflected by the fact that nine out of 22 autistic children were not able to complete the psychophysical task. The representativeness of our sample was better for our electrophysiological task, a passive task not involving a behavioral response. 

It is also worth mentioning that important developmental changes occur during the school age years with respect to FO and SO visual functions. In the present study, data was averaged across a wide range of ages (6 to 11 years-old) and consequently, the variability within each group may have masked subtle group differences. Nevertheless, analysis of covariance (ANCOVA) with chronological age as a covariate did not modify our negative behavioural findings for both FO and SO thresholds. Unfortunately, we could not perform reliable VEP analyses as a function of age, as assumptions for ANCOVA were often not met. There is thus a need for replication with larger samples.

Another methodological limitation is the use of only one level of modulation depth per condition during the electrophysiological task (6% for FO; 100% for SO). Depth modulations closer to threshold values may have been more favorable to highlight group differences, but higher contrasts were nonetheless necessary to elicit reliable cortical responses [52]. Studying a wider range of modulation depths in EEG would also have been more informative, but would have been difficult with regards to the amount of testing time needed to do so, considering the participants’ limited attention span. 

Importantly, current results in children must be interpreted in light of the fact that VEPs were recorded under a passive viewing condition. Late VEP components, particularly after 250-300 ms, are believed to be highly sensitive to the attentional demands of the task [52], but our experimental paradigm does not allow us to test if group differences arise from attentional factors. Any attentional differences between autistic and typical children most likely do not involve the allocation of overt attention, as two experimenters closely monitored the children’s behaviour during EEG acquisition. However, the role of covert attention is unknown, that is, shifts of the focus of attention in the absence of eye movements. Nevertheless, it is improbable that changes in covert attention selectively targeted texture but not luminance gratings, given the fact that both types of gratings were presented together in a pseudo-random sequence. Interactions between visual processing and attention are complex and profound, especially considering that they often share the same neural mechanisms [70]. Future studies on this topic should be aimed at further examining the influence of different attentional demands on the processing of different types of visual attributes (e.g., luminance, texture, color, movement). We know from studies on neurotypical observers that attention differently modulates brain responses depending on the visual attribute (see for example Di Russo et al., 2001 [71]), but the question remains to be addressed in autism.

Lastly, our exploratory correlational analyses revealed some between-group differences regarding the relationships between cognitive ability and FO-SO visual processes. The autism group, unlike our control group and other neurotypical observers [72], did not show a relationship between general fluid intelligence and sensitivity to texture stimuli. This is in line with other accounts of atypical relationships between lower-level perception and reasoning skills in autism [73]. Future research in the field of autism should further disentangle these relationships. Our preliminary results provide evidence that texture perception may be more pertinent than luminance perception to study in relation to intelligence and other higher level cognitive functions. 

## References

[1] Dakin S, Frith U (2005) Vagaries of visual perception in autism. Neuron 48: 497-507. doi:10.1016/j.neuron.2005.10.018. PubMed: 16269366. 16269366

[2] Simmons DR, Robertson AE, McKay LS, Toal E, McAleer P et al. (2009) Vision in autism spectrum disorders. Vision Res 49: 2705-2739. doi:10.1016/j.visres.2009.08.005. PubMed: 19682485. 19682485

[3] Wang L, Mottron L, Peng D, Berthiaume C, Dawson M (2007) Local bias and local-to-global interference without global deficit: a robust finding in autism under various conditions of attention, exposure time, and visual angle. Cogn Neuropsychol 24: 550-574. doi:10.1080/13546800701417096. PubMed: 18416507. 18416507

[4] Jarrold C, Gilchrist ID, Bender A (2005) Embedded figures detection in autism and typical development: preliminary evidence of a double dissociation in relationships with visual search. Dev Sci 8: 344-351. doi:10.1111/j.1467-7687.2005.00422.x. PubMed: 15985068. 15985068

[5] Shah A, Frith U (1983) An islet of ability in autism: a research note. J Child Psychol Psychiatry 24: 613-620. doi:10.1111/j.1469-7610.1983.tb00137.x. PubMed: 6630333. 6630333

[6] Caron MJ, Mottron L, Berthiaume C, Dawson M (2006) Cognitive mechanisms, specificity and neural underpinnings of visuospatial peaks in autism. Brain 129: 1789-1802. doi:10.1093/brain/awl072. PubMed: 16597652. 16597652

[7] Shah A, Frith U (1993) Why do autistic individuals show superior performance on the block design task? J Child Psychol Psychiatry 34: 1351-1364. doi:10.1111/j.1469-7610.1993.tb02095.x. PubMed: 8294523. 8294523

[8] Joseph RM, Keehn B, Connolly C, Wolfe JM, Horowitz TS (2009) Why is visual search superior in autism spectrum disorder? Dev Sci 12: 1083-1096. doi:10.1111/j.1467-7687.2009.00855.x. PubMed: 19840062. 19840062 PMC12049234

[9] Kaldy Z, Kraper C, Carter AS, Blaser E (2011) Toddlers with autism spectrum disorder are more successful at visual search than typically developing toddlers. Dev Sci 14: 980-988. doi:10.1111/j.1467-7687.2011.01053.x. PubMed: 21884314. 21884314 PMC3177163

[10] O’Riordan M, Plaisted K (2001) Enhanced discrimination in autism. Q J Exp Psychol A 54: 961-979. PubMed: 11765744. 11765744 10.1080/713756000

[11] Plaisted K, O’Riordan M, Baron-Cohen S (1998) Enhanced visual search for a conjunctive target in autism: a research note. J Child Psychol Psychiatry 39: 777-783. doi:10.1017/S0021963098002613. PubMed: 9690940. 9690940

[12] Happé F, Frith U (2006) The weak coherence account: Detail-focused cognitive style in autism spectrum disorders. J Autism Dev Disord 36: 1-21. doi:10.1007/s10803-005-0048-z. PubMed: 16450045. 16450045

[13] Mottron L, Bouvet L, Bonnel A, Samson F, Burack JA et al. (2013) Veridical mapping in the development of exceptional autistic abilities. Neurosci Biobehav Rev 37: 209-228. doi:10.1016/j.neubiorev.2012.11.016. PubMed: 23219745. 23219745

[14] Mottron L, Dawson M, Soulières I, Hubert B, Burack J (2006) Enhanced perceptual functioning in autism: an update, and 8 principles of autistic perception. J Autism Dev Disord 36: 27-43. doi:10.1007/s10803-005-0040-7. PubMed: 16453071. 16453071

[15] Braddick O, Atkinson J, Wattam-Bell J (2003) Normal and anomalous development of visual motion processing: motion coherence and ‘dorsal-stream vulnerability’. Neuropsychologia 41: 1769-1784. doi:10.1016/S0028-3932(03)00178-7. PubMed: 14527540. 14527540

[16] Davis RAO, Bockbrader MA, Murphy RR, Hetrick WP, O’Donnell BF (2006) Subjective perceptual distortions and visual dysfunction in children with autism. J Autism Dev Disord 36: 199-210. doi:10.1007/s10803-005-0055-0. PubMed: 16453070. 16453070

[17] Milne E, Swettenham J, Hansen P, Campbell R, Jeffries H et al. (2002) High motion coherence thresholds in children with autism. J Child Psychol Psychiatry 43: 255-263. doi:10.1111/1469-7610.00018. PubMed: 11902604. 11902604

[18] Spencer JV, O’Brien JM (2006) Visual form-processing deficits in autism. Perception 35: 1047-1055. doi:10.1068/p5328. PubMed: 17076065. 17076065

[19] Tsermentseli S, O’Brien JM, Spencer JV (2008) Comparison of form and motion coherence processing in autistic spectrum disorders and dyslexia. J Autism Dev Disord 38: 1201-1210. doi:10.1007/s10803-007-0500-3. PubMed: 18034294. 18034294

[20] Blake R, Turner LM, Smoski MJ, Pozdol SL, Stone WL (2003) Visual recognition of biological motion is impaired in children with autism. Psychol Sci 14: 151-157. doi:10.1111/1467-9280.01434. PubMed: 12661677. 12661677

[21] Del Viva MM, Igliozzi R, Tancredi R, Brizzolara D (2006) Spatial and motion integration in children with autism. Vision Res 46: 1242-1252. doi:10.1016/j.visres.2005.10.018. PubMed: 16384591. 16384591

[22] Vandenbroucke MW, Scholte HS, van Engeland H, Lamme VAF, Kemner C (2008) Coherent versus component motion perception in autism spectrum disorder. J Autism Dev Disord 38: 941-949. doi:10.1007/s10803-007-0467-0. PubMed: 17952582. 17952582

[23] Baker CL Jr, Mareschal I (2001) Processing of second-order stimuli in the visual cortex. Prog Brain Res 134: 171-191. doi:10.1016/S0079-6123(01)34013-X. PubMed: 11702543. 11702543

[24] Landy MS, Graham N (2004) Visual perception of texture. In: Chalupa LM Werner JS The visual Neurosciences. Cambridge, MA: MIT Press pp. 1106-1118.

[25] Chubb C, Sperling G (1988) Drift-balanced random stimuli: a general basis for studying non-Fourier motion perception. J Opt Soc Am A 5: 1986-2007. doi:10.1364/JOSAA.5.001986. PubMed: 3210090. 3210090

[26] Ashida H, Lingnau A, Wall MB, Smith AT (2007) FMRI adaptation reveals separate mechanisms for first-order and second-order motion. J Neurophysiol 97: 1319-1325. PubMed: 17065251. 17065251 10.1152/jn.00723.2006

[27] Zhou YX, Baker CL Jr (1994) Envelope-responsive neurons in areas 17 and 18 of cat. J Neurophysiol 72: 2134-2150. PubMed: 7884449. 7884449 10.1152/jn.1994.72.5.2134

[28] Calvert J, Manahilov V, Simpson WA, Parker DM (2005) Human cortical responses to contrast modulations of visual noise. Vision Res 45: 2218-2230. doi:10.1016/j.visres.2005.02.012. PubMed: 15924937. 15924937

[29] Ellemberg D, Lavoie K, Lewis TL, Maurer D, Lepore F et al. (2003) Longer VEP latencies and slower reaction times to the onset of second-order motion than to the onset of first-order motion. Vision Res 43: 651-658. doi:10.1016/S0042-6989(03)00006-3. PubMed: 12604101. 12604101

[30] Vaina LM, Cowey A (1996) Impairment of the perception of second order motion but not first order motion in a patient with unilateral focal brain damage. Proc Biol Sci 263: 1225-1232. doi:10.1098/rspb.1996.0180. PubMed: 8858874. 8858874

[31] Vaina LM, Makris N, Kennedy D, Cowey A (1998) The selective impairment of the perception of first-order motion by unilateral cortical brain damage. Vis Neurosci 15: 333-348. PubMed: 9605533. 9605533 10.1017/s0952523898152082

[32] Dumoulin SO, Baker CL Jr, Hess RF, Evans AC (2003) Cortical specialization for processing first- and second-order motion. Cereb Cortex 13: 1375-1385. doi:10.1093/cercor/bhg085. PubMed: 14615303. 14615303

[33] Larsson J, Landy MS, Heeger DJ (2006) Orientation-selective adaptation to first- and second-order patterns in human visual cortex. J Neurophysiol 95: 862-881. PubMed: 16221748. 16221748 10.1152/jn.00668.2005 PMC1538978

[34] Smith AT, Greenlee MW, Singh KD, Kraemer FM, Hennig J (1998) The processing of first- and second-order motion in human visual cortex assessed by functional magnetic resonance imaging (fMRI). J Neurosci 18: 3816-3830. PubMed: 9570811. 9570811 10.1523/JNEUROSCI.18-10-03816.1998 PMC6793149

[35] Bertone A, Mottron L, Jelenic P, Faubert J (2003) Motion perception in autism: A “complex” issue. J Cogn Neurosci 15: 218-225. doi:10.1162/089892903321208150. PubMed: 12676059. 12676059

[36] Bertone A, Mottron L, Jelenic P, Faubert J (2005) Enhanced and diminished visuo-spatial information processing in autism depends on stimulus complexity. Brain 128: 2430-2441. doi:10.1093/brain/awh561. PubMed: 15958508. 15958508

[37] Kogan CS, Bertone A, Cornish K, Boutet I, Der Kaloustian VM, et al. (2004) Integrative cortical dysfunction and pervasive motion perception deficit in fragile X syndrome. Neurology 63: 1634-1639 10.1212/01.wnl.0000142987.44035.3b 15534248

[38] Brosseau-Lachaine O, Gagnon I, Forget R, Faubert J (2008) Mild traumatic brain injury induces prolonged visual processing deficits in children. Brain Inj 22: 657-668. doi:10.1080/02699050802203353. PubMed: 18698516. 18698516

[39] Lachapelle J, Ouimet C, Bach M, Ptito A, Mckerral M (2004) Texture segregation in traumatic brain injury- a VEP study. Vision Res 44: 2835-2842. doi:10.1016/j.visres.2004.06.007. PubMed: 15342227. 15342227

[40] Habak C, Faubert J (2000) Larger effect of aging on the perception of higher-order stimuli. Vision Res 40: 943-950. doi:10.1016/S0042-6989(99)00235-7. PubMed: 10720665. 10720665

[41] Bertone A, Faubert J (2006) Demonstrations of decreased sensitivity to complex motion information not enough to propose an autism-specific neural etiology. J Autism Dev Disord 36: 55-64. doi:10.1007/s10803-005-0042-5. PubMed: 16374669. 16374669

[42] Kéïta L, Mottron L, Dawson M, Bertone A (2011) Atypical lateral connectivity: a neural basis for altered visuospatial processing in autism. Biol Psychiatry 70: 806-811. doi:10.1016/j.biopsych.2011.07.031. PubMed: 21907325. 21907325

[43] Kemner C, Lamme VAF, Kovacs I, van Engeland H (2007) Integrity of lateral and feedbackward connections in visual processing in children with pervasive developmental disorder. Neuropsychologia 45: 1293-1298. doi:10.1016/j.neuropsychologia.2006.09.016. PubMed: 17101159. 17101159

[44] Armstrong V, Maurer D, Lewis TL (2009) Sensitivity to first- and second-order motion and form in children and adults. Vision Res 49: 2774-2781. doi:10.1016/j.visres.2009.08.016. PubMed: 19699227. 19699227

[45] Bertone A, Hanck J, Cornish KM, Faubert J (2008) Development of static and dynamic perception for luminance-defined and texture-defined information. Neuroreport 19: 225-228. doi:10.1097/WNR.0b013e3282f48401. PubMed: 18185113. 18185113

[46] Lord C, Rutter M, Le Couteur A (1994) Autism Diagnostic Interview-Revised: a revised version of a diagnostic interview for caregivers of individuals with possible pervasive developmental disorders J Autism Dev Disord 24: 659-685. doi:10.1007/BF02172145. PubMed: 7814313. 7814313

[47] Lord C, Rutter M, Goode S, Heemsbergen J, Jordan H et al. (1989) Autism diagnostic observation schedule: a standardized observation of communicative and social behaviour. J Autism Dev Disord 19: 185-212. doi:10.1007/BF02211841. PubMed: 2745388. 2745388

[48] Raven J, Raven JC, Court JH (1998) Raven manual: Section 3. Standard progressive matrices. Oxford, England: Oxford Psychologists Press. 106 pp.

[49] Dunn LM, Dunn LM (1981) Peabody Picture Vocabulary Test-Revised. Circle Pines, MN: American Guidance Service. 175 p.

[50] Hyvärinen L, Näsänen R, Laurinen P (1980) New visual acuity test for pre-school children. Acta Ophthalmol 58: 507-511. PubMed: 7211248. 7211248 10.1111/j.1755-3768.1980.tb08291.x

[51] Smith AT, Ledgeway T (1997) Separate detection of moving luminance and contrast modulations: fact or artifact? Vision Res 37: 45-62. doi:10.1016/S0042-6989(96)00147-2. PubMed: 9068830. 9068830

[52] Luck SJ (2005) An introduction to the event-related potential technique. Cambridge, MA: MIT Press. 374 pp.

[53] Tucker DM (1993) Spatial sampling of head electrical fields: the geodesic sensor net. Electroencephalogr Clin Neurophysiol 87: 154-163. doi:10.1016/0013-4694(93)90121-B. PubMed: 7691542. 7691542

[54] Nunez PL (1981) Electric fields of the brain: The neurophysics of EEG. New York: Oxford University Press. 484 pp.

[55] Gratton G, Coles MG, Donchin E (1983) A new method for off-line removal of ocular artifact. Electroencephalogr Clin Neurophysiol 55: 468–484. doi:10.1016/0013-4694(83)90135-9. PubMed: 6187540. 6187540

[56] Pernier J, Perrin F, Bertrand O (1988) Scalp current density fields: Concept and properties. Electroencephalogr Clin Neurophysiol 69: 385-389. doi:10.1016/0013-4694(88)90009-0. PubMed: 2450736. 2450736

[57] Perrin F, Pernier J, Bertrand O, Echallier JF (1989) Spherical splines for scalp potential and current density mapping. Electroencephalogr Clin Neurophysiol 72: 184-187. doi:10.1016/0013-4694(89)90180-6. PubMed: 2464490. 2464490

[58] Kayser J (2009) Current source density (CSD) interpolation using spherical splines - CSD Toolbox (Version 1.1). New York State Psychiatric Institute: Division of Cognitive Neuroscience. Available: http://psychophysiology.cpmc.columbia.edu /Software/CSDtoolbox. Accessed March 30, 2013.

[59] Arcand C, Tremblay E, Vannasing P, Ouimet C, Roy MS et al. (2007) Development of visual texture segregation during the first year of life: a high-density electrophysiological study. Exp Brain Res 180: 263-272. doi:10.1007/s00221-007-0854-y. PubMed: 17265040. 17265040

[60] Clark VP, Fan S, Hillyard SA (1995) Identification of early visual evoked potential generators by retinotopic and topographic analyses. Hum Brain Mapp 2: 170-187.

[61] Di Russo F, Pitzalis S, Spitoni G, Aprile T, Patria F et al. (2005) Identification of the neural sources of the pattern-reversal VEP. NeuroImage 24: 874-886. doi:10.1016/j.neuroimage.2004.09.029. PubMed: 15652322. 15652322

[62] Bullier J (2001) Integrated model of visual processing. Brain. Res Rev 36: 96-107. doi:10.1016/S0165-0173(01)00085-6. 11690606

[63] Boeschoten MA, Kenemans JL, van Engeland H, Kemner C (2007) Abnormal spatial frequency processing in high-functioning children with pervasive developmental disorder (PDD). Clin Neurophysiol 118: 2076-2088. doi:10.1016/j.clinph.2007.05.004. PubMed: 17591458. 17591458

[64] Jemel B, Mimeault D, Saint-Amour D, Hosein A, Mottron L (2010) VEP contrast sensitivity responses reveal reduced functional segregation of mid and high filters of visual channels in autism. J Vis 10: 13. doi:10.1167/10.10.13. PubMed: 20884562. 20884562

[65] Milne E, Scope A, Pascalis O, Buckley D, Makeig S (2009) Independent component analysis reveals atypical electroencephalographic activity during visual perception in individuals with autism. Biol Psychiatry 65: 22-30. doi:10.1016/j.biopsych.2008.07.017. PubMed: 18774554. 18774554

[66] Greenaway R, Davis G, Plaisted-Grant K (2013) Marked selective impairment in autism on an index of magnocellular function. Neuropsychologia 51: 592-600. doi:10.1016/j.neuropsychologia.2013.01.005. PubMed: 23333905. 23333905

[67] Vandenbroucke MW, Scholte HS, van Engeland H, Lamme VAF, Kemner C (2008) A neural substrate for atypical low-level visual processing in autism spectrum disorder. Brain 131: 1013-1024. doi:10.1093/brain/awm321. PubMed: 18192288. 18192288

[68] Barlow HB (1972) Single units and sensation: a neuron doctrine for perceptual psychology? Perception 1: 371-394. doi:10.1068/p010371. PubMed: 4377168. 4377168

[69] Lewis TL, Kingdon A, Ellemberg D, Maurer D (2007) Orientation discrimination in 5-year-olds and adults tested with luminance-modulated and contrast-modulated gratings. J Vis 7: 9. doi:10.1167/7.7.9. PubMed: 17461693. 17461693

[70] Treue S (2001) Neural correlates of attention in primate visual cortex. Trends Neurosci 24: 295-300. doi:10.1016/S0166-2236(00)01814-2. PubMed: 11311383. 11311383

[71] Di Russo F, Spinelli D, Morrone MC (2001) Automatic gain control contrast mechanisms are modulated by attention in humans: evidence from visual evoked potentials. Vision Res 41: 2435-2447. doi:10.1016/S0042-6989(01)00134-1. PubMed: 11483175. 11483175

[72] Jolij J, Huisman D, Scholte S, Hamel R, Kemner C, Lamme VAF (2007) Processing speed in recurrent visual networks correlates with general intelligence. Neuroreport 18: 39-43. doi:10.1097/01.wnr.0000236863.46952.a6. PubMed: 17259858. 17259858

[73] Liu Y, Cherkassky VL, Minshew NJ, Just MA (2011) Autonomy of lower-level perception from global processing in autism: Evidence from brain activation and functional connectivity. Neuropsychologia 49: 2105-2111. doi:10.1016/j.neuropsychologia.2011.04.005. PubMed: 21513720. 21513720 PMC4500152

