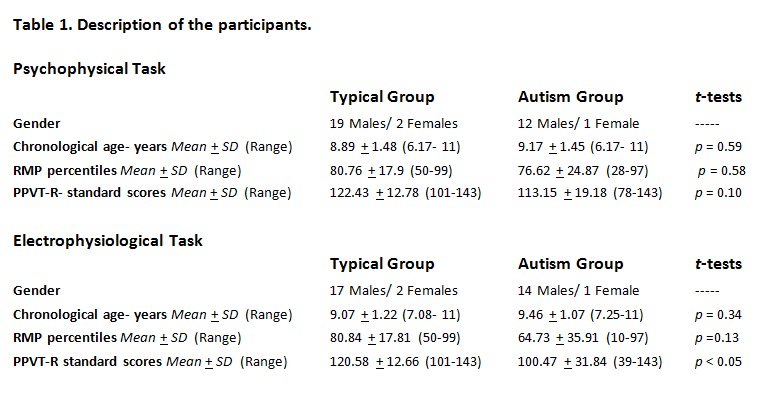# Correction: Luminance- and Texture-Defined Information Processing in School-Aged Children with Autism

**DOI:** 10.1371/annotation/a4b3468f-cb36-4833-85f9-93a7cba7c36a

**Published:** 2013-11-12

**Authors:** Jessica B. Rivest, Boutheina Jemel, Armando Bertone, Michelle McKerral, Laurent Mottron

The heading "Electrophysiological Task" in Table 1 was misplaced into the table legend. It should appear above the lower half of the participant groups. Please see the correct Table 1 here: 

**Figure:**